# Intestinal overexpression of *Pla2g10* alters the composition, diversity and function of gut microbiota in mice

**DOI:** 10.3389/fcimb.2025.1535204

**Published:** 2025-03-14

**Authors:** Wenhao Liao, Lei Cao, Xuemei Jiang, Lianqiang Che, Zhengfeng Fang, Shengyu Xu, Yan Lin, Yong Zhuo, Lun Hua, Jian Li, Guangmang Liu, Mengmeng Sun, De Wu, Hairui Wang, Bin Feng

**Affiliations:** ^1^ Animal Nutrition Institute, Sichuan Agricultural University, Chengdu, Sichuan, China; ^2^ Key Laboratory of Animal Disease-Resistant Nutrition of Sichuan Province, Sichuan Agricultural University, Chengdu, Sichuan, China; ^3^ College of Science, Sichuan Agricultural University, Ya’an, Sichuan, China; ^4^ Chengdu Research Base of Giant Panda Breeding, Sichuan Key Laboratory of Conservation Biology for Endangered Wildlife, Chengdu, China

**Keywords:** PLA2G10, gut microbiota, intestine, *Allobaculum*, *Dubosiella*

## Abstract

The intestinal microbiota is important for the health of the host and recent studies have shown that some genes of the host regulated the composition of the intestinal microbiota. Group 10 phospholipase A2 (PLA2G10) is a member of the lipolytic enzyme family PLA2, which hydrolyze the ester bond at the sn-2 position of phospholipids to produce free fatty acids and lysophospholipids. PLA2G10 is secreted into the intestinal lumen, but its impact on the gut microbiota remains unclear. In this study, we generated intestine-specific *Pla2g10* knock-in mice, and used 16S RNA sequencing to compare their gut microbiota with that of their wild-type (WT) littermates. Results showed that gut-specific *Pla2g10* knock-in induced both PLA2G10 mRNA and protein levels in the colon. Moreover, intestinal *Pla2g10* overexpression reduced the α-diversity of the gut microbiota relative to that of WT mice. The abundance of Bacteroidetes was lower in the *Pla2g10* knock-in mice than that in the control mice, while the ratio of Firmicutes/Bacteroidetes was higher. Furthermore, the abundance of the genus *Allobaculum* was reduced, whereas the abundance of beneficial bacteria genera, including *Enterorhabdus*, *Dubosiella*, and *Lactobacillus*, was increased by host intestinal *Pla2g10* overexpression. In summary, intestinal *Pla2g10* overexpression increased the proportions of beneficial bacterial in the colonic chyme of mice, providing a potential therapeutic target for future improvement of the gut microbiota.

## 1 Introduction

In recent years, our understanding of the gut microbiota has greatly improved. Our gastrointestinal tract, particularly the colon, contains a great variety of microbes, harbors a wide variety of bacteria, viruses and other microorganisms. These gut microbes interact with the host’s epithelial and immune cells, forming a biological barrier in the gut and coordinating the primary and secondary metabolism on the surface of the intestinal mucosa (Durack and Lynch, 2019). In addition, the gut microbiota coexists symbiotically with its host, participating in numerous physiological activities and playing crucial roles in the host’s health and the development of diseases. The numbers, species, abundance, and evenness of the microorganisms that make up this complex ecosystem affect the host’s health, and the ecosystem is in a state of dynamic homeostasis, and any disruption of its balance increases the risk of disease in the host (Chen et al., 2021; Clemente et al., 2018; Durack and Lynch, 2019).

The phospholipase A2 (PLA2) family belongs to the lipolytic enzymes and its members hydrolyze the sn-2 ester bond of phospholipids to produce free fatty acids and lysophospholipids. Secretory PLA2 (sPLA2) constitutes the largest subfamily of PLA2, with 10 catalytically active sPLA2s identified in mammals: PLA2G1B, -2A, -2C, -2D, -2E, -2F, -3, -5, -10, and -12 (Dennis et al., 2011). Among the secretory phospholipases, PLA2G10 contains a histidine/asparagine catalytic dyad and a highly conserved Ca^2+^-binding loop, and conserved disulfide bonds contribute to its structural stability (Murakami et al., 2023). PLA2G10 is synthesized as a zymogen and converted into an active mature enzyme by the proteolytic cleavage of the N-terminal propeptide. This processing can occur within the cell before its secretion, by protease-like conversion enzymes, or outside the cell after its secretion, as is the case for various digestive enzymes in the gastrointestinal tract (Jemel et al., 2011; Layne et al., 2015; Masuda et al., 2005).

PLA2G10 is highly expressed in the Paneth cells of the colon (Schewe et al., 2016) and it has a greater capacity than other PLAs to hydrolyze phosphatidylcholine (PC), the major phospholipid component of the plasma membrane, producing lysophosphatidylcholine (LPC) and free fatty acids (such as polyunsaturated fatty acids of arachidonic acid, eicosapentaenoic acid, docosapentaenoic acid and docosahexaenoic acid) (Bezzine et al., 2000; Hanasaki et al., 1999; Kudo et al., 2022; Murase et al., 2016). Reports have suggested that dietary supplementation of polyunsaturated fatty acids could change the diversity and abundance of gut microbiota (Cao et al., 2019; Feng et al., 2020; Mujico et al., 2013; Tang et al., 2021; Zhuang et al., 2017). Additional, several studies have demonstrated that various secretory phospholipases alter the composition and abundance of the gut microbiota in mice. For instance, the relative abundance of *Bacteroides* correlates positively with the expression of *Pla2g5*, *Pla2g2a*, and *Pla2g2f*, whereas the abundances of *norank_f_ Erysipelotrichaceae* and *Roseburia* correlate negatively with these three enzymes. *Pla2g2a* ^−/−^ mice have reduced *Coriobacteriaceae FCS020* and *Ruminococcaceae UCG-013*, whereas *Coriobacteriaceae UCG-008* is significantly elevated compared with its abundance in *Pla2g2a* ^+/+^ mice (Taketomi et al., 2022; Zhang et al., 2023). However, the researches on the effect of *Pla2g10* on gut microbiota are still few. To further investigate the effect of intestinal *Pla2g10* overexpression on gut microbiota, we generated intestine-specific *Pla2g10* knock-in mice, which specifically overexpressed *Pla2g10* in intestine. Results showed that overexpression of *Pla2g10* in intestine altered the composition, diversity and function of the gut microbiota in mice.

## 2 Materials and methods

### 2.1 Animals

**Figure 1:**
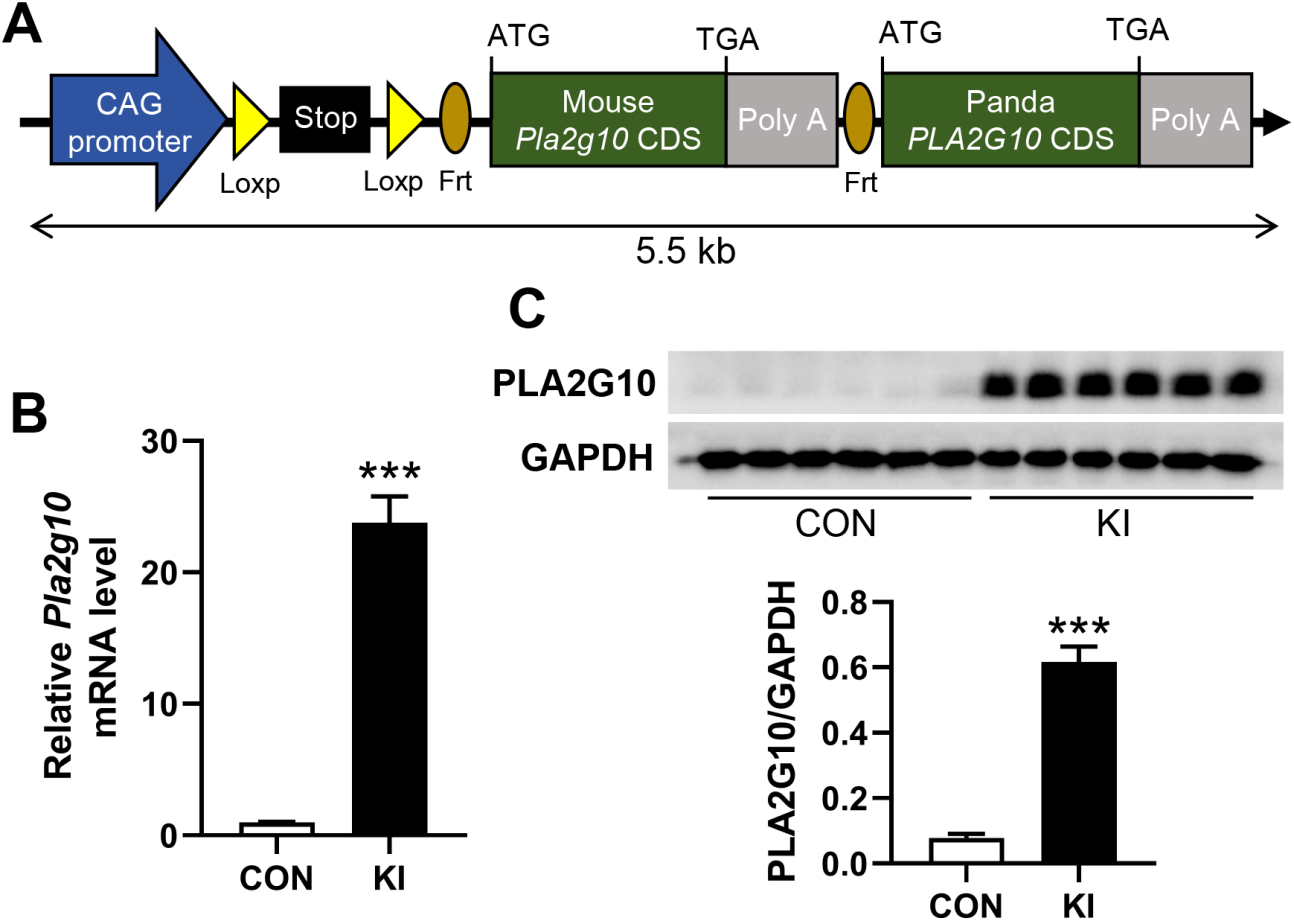
Generation of intestine-specific *Pla2g10* knock-in mice. **(A)** Schematic for construction of mouse *Pla2g10* loxp mice. Coding sequence of panda *PLA2G10* was inserted for future study. **(B)** qPCR analysis for *Pla2g10* gene expression in mice colon (n = 6). **(C)** Western blot analysis for PLA2G10 protein expression in mice colon. Data is presented as mean ± SEM. CON, control group; KI, *Pla2g10* konck-in group. ***P < 0.001.

All animal protocols were reviewed and approved by the Animal Care and Use Committee of Sichuan Agricultural University (SICAU-2020-280, 12 May 2020). All animal studies were performed in accordance with the Use and Care of Laboratory Animals. *Pla2g10* ^loxp+/−^ mice were constructed with C57BL/6 background Embryonic stem cells (**Figure 1A**). C57BL/6J-background tomoxifen-dependent *Lgr5* ^cre+^ mice (T003768) were purchased from GemPharmatech LLC., China. *Pla2g10* knock-in (KI, *Pla2g10* ^loxp+/−^ *Lgr5* ^cre+/−^, n = 6) mice were generated by mating *Pla2g10* ^loxp+/−^ mice with *Lgr5* ^cre+^ mice, and *Pla2g10* ^loxp+/−^ mice (n = 6) were used as the control. All the mice were housed under the individual ventilated caging system, maintained in a 12-h light/12-h dark environment, with a room temperature maintained of 22°C and humidity of 60%, with free access to food and water.

Mice with the same genotype were housed together, with 2 mice per cage. At an age of 4 months, the mice were weighted and injected with 100 mg/kg tamoxifen (#S1238, Selleck, Shanghai, China) once daily for a week to induce Cre expression. After injection, their body weight were recorded weekly for 4 weeks, and fed blood glucose levels were measured twice weekly, and food intake were recorded daily for 4 days. 4 weeks after tamoxifen injection, mice were euthanized with CO_2_. The colons were collected, and the chyme in the colons was collected for microbial DNA extraction.

### 2.2 Food intake

The initial weight of food in each cage on the first day was recorded at 5 pm, while the weight of the remaining food was measured at 5 pm on the following days. The food intake was calculated as (initial food weight - remaining food weight)/2.

### 2.3 Real-time qPCR

Total RNA was extracted from tissues using RNAiso Plus (#9109, TaKaRa Bio, Dalian, China) following the manufacturer’s instructions. 1 μg of total RNA was used to synthesize cDNA using the PrimeScript FAST RT reagent Kit with gDNA Eraser (#RR092A, TaKaRa Bio). The cDNA was then subjected to qPCR using the iTaq Universal SYBR Green Supermix (#1725124, Bio-Rad, Shanghai, China) with a 384-well real-time PCR machine (QuantStudio 5, Thermo Fisher, Shanghai, China). The following primers were used: *Gapdh* F: 5’-AGGTCGGTGTGAACGGATTTG-3′, R: 5′-TGTAGACCATGTAGTTGAGGTCA-3′; *Pla2g10* F: 5’-GTGCAGGTGTGACGAGGAG-3′, R:5′-CACTTGGGAGAGTCCTTCTCA-3′.

### 2.4 Western blotting

After homogenized in Western ReProbe (TM) PLUS (#C500035, Sangon Biotech, Shanghai, China) supplemented with PMSF (#abs812852, Absin, Shanghai, China), the protein samples were centrifuged at 12,000 rpm for 30 min. The protein lysates were quantified using a BCA protein assay kit (#23227, Thermo Fisher, Shanghai, China). Protein samples with equivalent concentrations were separated by SDS-PAGE. The following antibodies were used: rabbit anti-GAPDH Ab (#60004-1-lg, Proteintech, Wuhan, China) and mouse anti-PLA2G10 Ab (#sc-514324, Santa cruz, Shanghai, China).

### 2.5 16s RNA sequencing

Genomic DNA (gDNA) was extracted from the colonic chyme using a soil genomic DNA extraction kit (#DP336, Tiangen biotech, Beijing, China). The gDNA was used as a template to amplify the highly-variable V3–V4 region of the 16S RNA gene by PCR, using the forward primer 341F (5’-ACTCCTACGGGAGGCAGCAG-3’) and the reverse primer 806R (5’-GGACTACHVGGGTWTCT AAT-3’). All PCRs were performed in 15μL of Phusion High-Fidelity PCR Master Mix (New England Biolabs, Manchester, UK), with 0.2 μM of both the forward and reverse primers, and approximately 10 ng of template DNA. Thermal cycling consisted of an initial denaturation at 98°C for 1 minute, followed by 30 cycles of denaturation at 98°C for 10s, annealing at 50°C for 30s, and elongation at 72°C for 30s, and finally an extension at 72°C for 5 min.

The PCR products were purified using magnetic beads. The samples were mixed in equal-density ratios based on the concentrations of the PCR products. After the PCR products were thoroughly mixed, they were detected and purified using a universal DNA purification and recovery kit (#DP214-03, Tiangen Biotech, Beijing, China) to isolate the nucleic acids. The nucleic acids were subjected to construct libraries using a library construction kit (#E7645S, New England BioLabs, Manchester, UK). The libraries were sequenced using the Illumina NovaSeq 6000 (PE250) system.

### 2.6 Sequence analysis

Paired-end reads were merged using the FLASH (v1.2.11) to obtain raw tags (Magoč and Salzberg, 2011). The Cutadapt software (v3.3) was then used to match the reverse primer sequence and trim the remaining sequences to prevent their interference in subsequent analyses. Quality filtering of the raw tags was performed using the fastp software (v0.23.1) to obtain high-quality clean tags (Bokulich et al., 2013). To detect chimeric sequences, the tags were compared with the reference database (Silva database (16S/18S), and the effective tags were obtained by removing the chimeric sequences using the vsearch package (v2.16.0) (Edgar et al., 2011). To obtain the initial amplicon sequence variants (ASVs), the DATA2 program in the QIIME2 software (v202202) was performed to denoise the previous effective tags (Y. Wang et al., 2021). The species annotation was performed with QIIME2 software.

The absolute abundance of ASVs was normalized using a standard of sequence number corresponding to the sample with the fewest sequences. The subsequent analyses of α-diversity and β-diversity were all performed based on the output normalized data. The top 10 taxa at the phylum level and top 22 taxa at the genus level of each sample were selected to plot the distribution histogram of relative abundance using Perl software (v5.26.2). The Venn diagram was produced in R software (v4.0.3) using the Venn Diagram function, and the α-diversity was calculated using Chao1 and Shannon indices in QIIME2 software to analyze the diversity, richness and uniformity. The β-diversity was calculated by the unweighted Unifrac distance using QIIME2 software, and was visualized by principal coordinate analysis (PCoA) and non-metric multidimensional scaling (NMDS) analysis using R software (v4.0.3). The Linear Discriminant Analysis Effect Size (LEfSe) was applied to analyze the differences at each level using R software (v4.0.3). The functional genes and Kyoto Encyclopedia of Genes and Genomes (KEGG) orthologs were predicted by the Phylogenetic Investigation of Communities by Reconstruction of Unobserved States (PICRUSt2) (v2.3.0). The network plot was produced using Chiplot (https://www.chiplot.online/).

### 2.7 Statistical analysis

Data are presented as mean ± standard error of the mean (SEM). Growth data were preliminarily analyzed using Microsoft Excel 2019 and then analyzed statistically using the SPSS 21.0 software. Normality of the data was evaluated by Shapiro-Wilk test using the SPSS 21.0 software. Microbial data were organized and analyzed statistically using the R software (v4.0.3). Data were visualized using the GraphPad Prism (v8.0) and R software (v4.0.3) The P - values used to compare two groups were calculated with Student’s *t*-test. A trend was deemed present when 0.05 < *P* < 0.1; the significance of differences was defined as *P < 0.05.

## 3 Results

### 3.1 Intestinal *Pla2g10* overexpression reduced the abundance of gut microbiota

**Figure 2:**
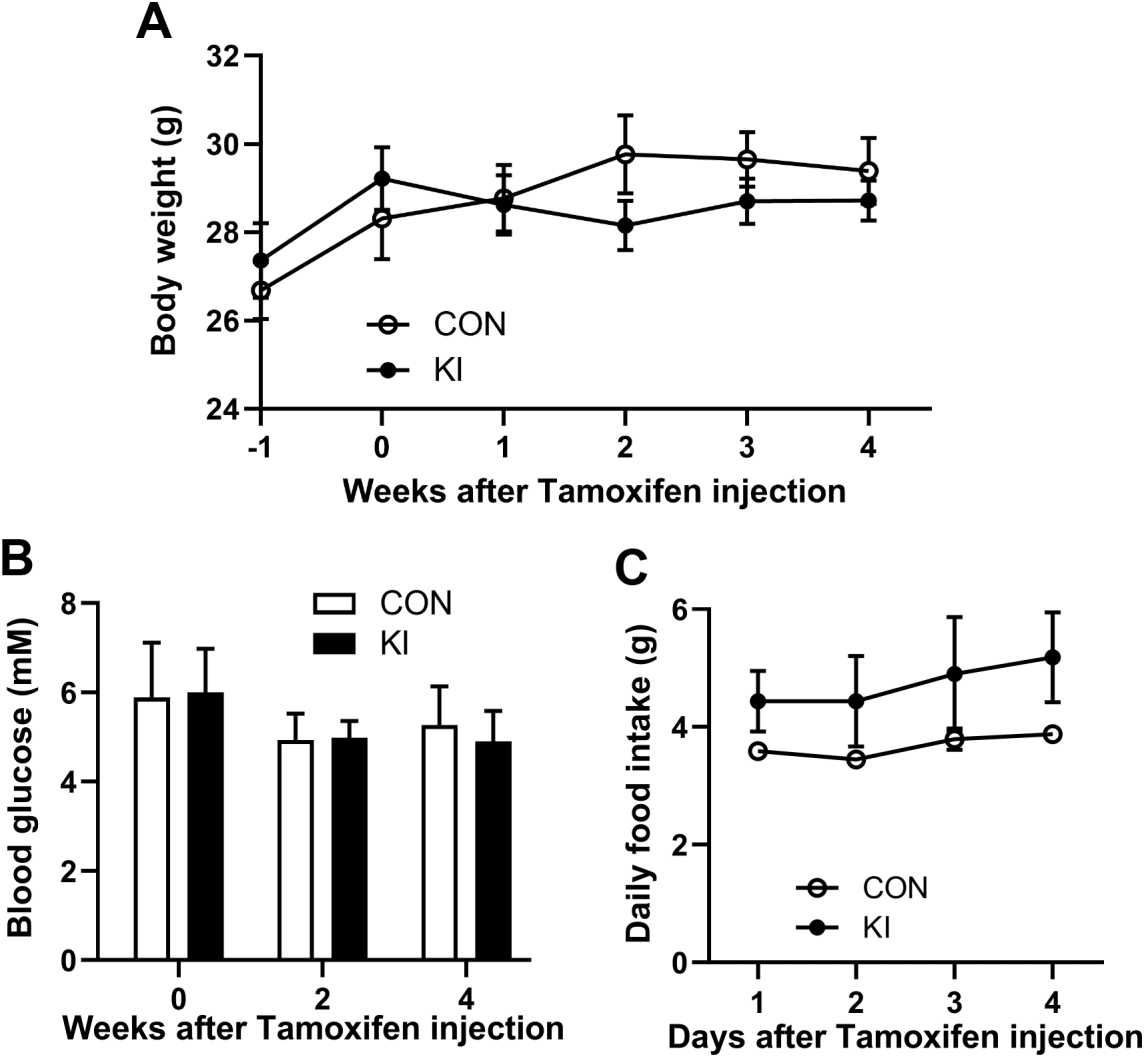
*Pla2g10* overexpression did not alter the body weight, food intake, and blood glucose levels of mice. **(A)** The body weight (n = 6). **(B)** Blood glucose levels (n = 6). **(C)** Food intake (n = 3). Mice with the same genotype were housed together, with 2 mice per cage. The initial weight of food in each cage on the first day was recorded at 5 pm, while the weight of the remaining food was measured at 5 pm on the following days. The food intake was calculated as (initial food weight - remaining food weight)/2. Data is presented as mean ± SEM. CON, control group; KI, *Pla2g10* konck-in group.

**Figure 3:**
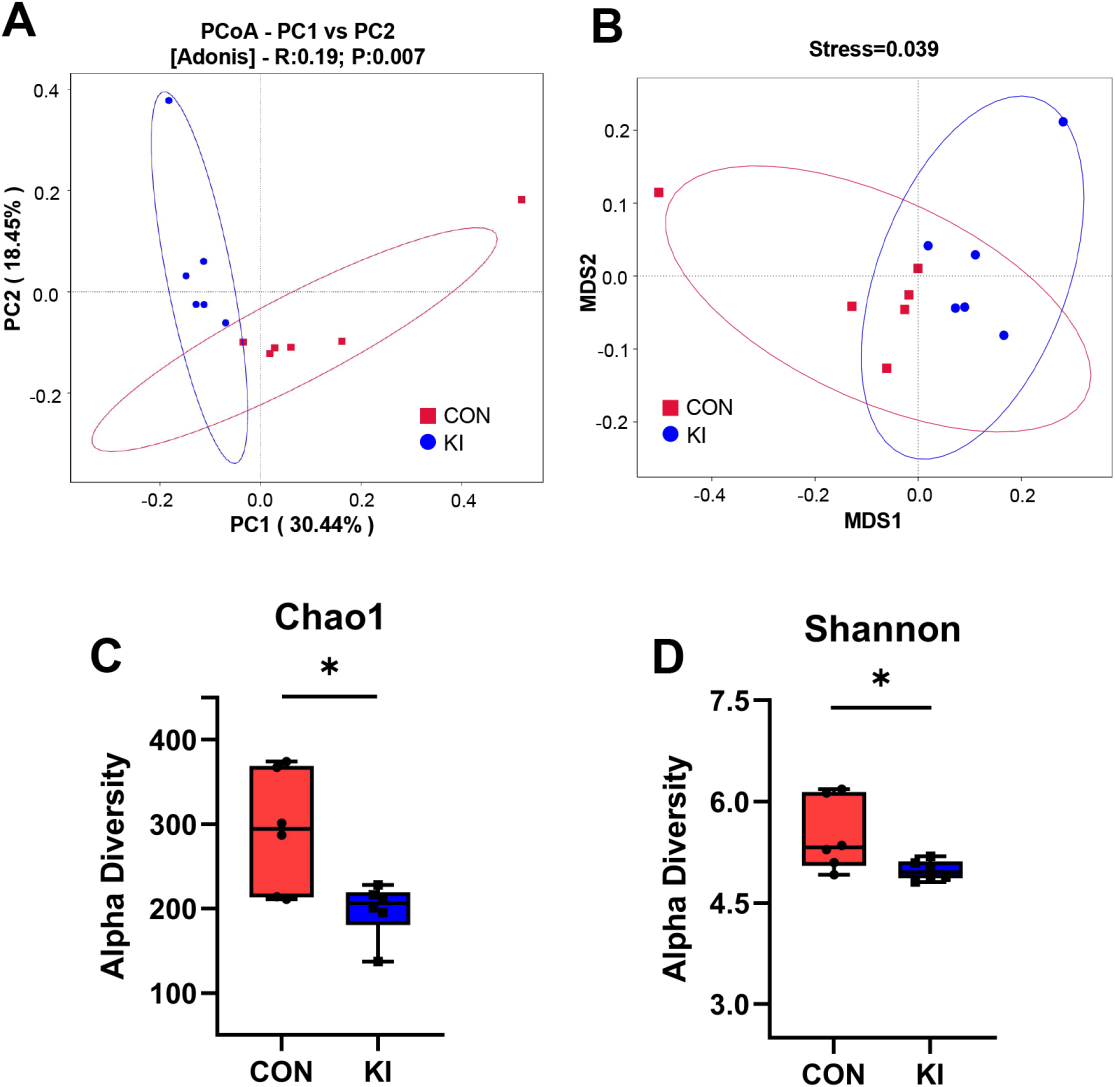
*Pla2g10* overexpression altered the composition of the gut microbiota in mice. **(A, B)** PCoA and NMDS plot, distance algorithm: unweighted_unifrac. **(C, D)** Shannon and Chao1 index of α-diversity. Data is presented as mean ± SEM. *P < 0.05. CON, control group; KI, *Pla2g10* konck-in group.

To explore the effects of intestinal *Pla2g10* overexpression on the homeostasis of gut microbiota, *Pla2g10* ^loxp+/−^ mice were mated with tamoxifen-dependent *Lgr5* ^cre+^ mice to generate intestine-specific *Pla2g10* knock-in (KI) mice. Both the gene expression and protein levels of PLA2G10 were significantly higher in the colons of the *Pla2g10* KI mice than in those of the control mice (**Figures 1B, C**). In the subsequent 4 weeks after CRE induced, the body weights and glucose levels did not differ significantly between the two groups (**Figures 2A, B**). Although there was no significant difference in the food intake of the two groups, the *Pla2g10* KI mice consistently consumed 1 g more than the control mice (**Figure 2C**). A PCoA with an unweighted UniFrac distance algorithm revealed differences in the microbial compositions of the two groups (**Figure 3A**). The first principal axis (PCoA1) could explain 30.44% of the sample difference, and the second principal axis (PCoA2) could explained 18.45% of the sample difference. The NMDS stress value was 0.039, indicating that the subsequent analytical results accurately reflected the differences between the samples with high confidence (**Figure 3B**). We assessed the α-diversity of the gut microbiota by calculating the Chao1 and Shannon indices, both of which were significantly lower in the *Pla2g10* KI mice than in the control group (**Figures 3C, D**), indicating that the total number of gut bacteria and their richness and evenness were reduced after the overexpression of *Pla2g10*. A Venn diagram confirmed this, showing that the number of operational taxonomic units (OTUs) was lower in the *Pla2g10* KI mice than in the control group (**Supplementary Figure 1A**).

### 3.2 Intestinal *Pla2g10* overexpression alters the composition of the gut microbiota

**Figure 4:**
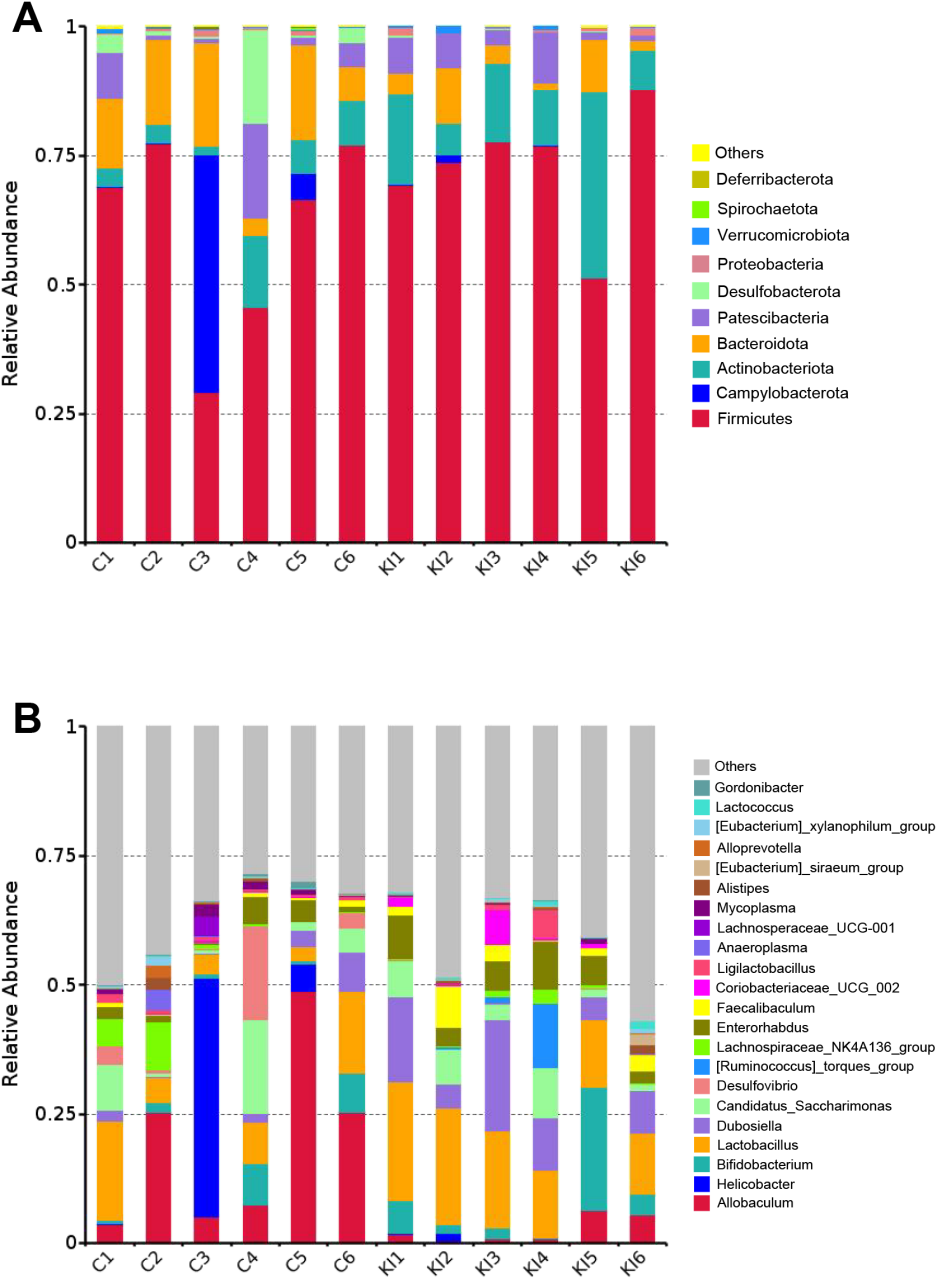
Taxonomic comparison of gut microbiota in *Pla2g10* KI and control mice. Taxonomic composition of the gut microbiota at the phylum **(A)** and genus **(B)** levels. C, control group; KI, *Pla2g10* konck-in group.

**Figure 5:**
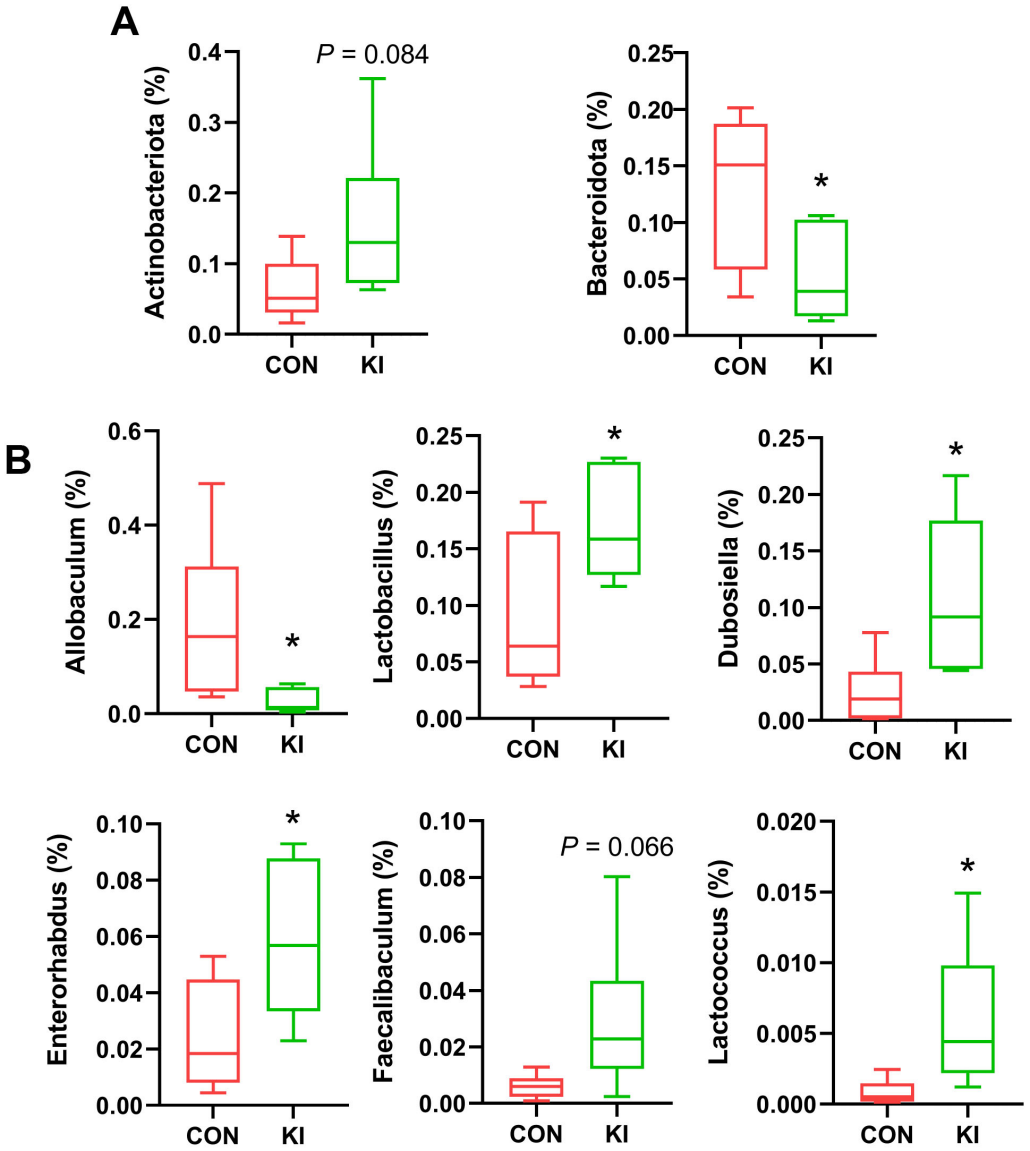
Bacterial taxa in the chyme of *Pla2g10* KI and control mice. Significantly changed bacterial taxa at the phylum **(A, B)** genus levels. Data is presented as mean ± SEM. CON, control group; KI, *Pla2g10* konck-in group. *P < 0.05.

Analysis of the community composition and species differences between the *Pla2g10* KI and control mice showed that the overexpression of *Pla2g10* altered the composition of the gut microbiota at the phylum (**Figure 4A**) and genus levels (**Figure 4B**). The detailed distributions of the gut microbiotal compositions of the two groups are shown in **Supplementary Table 1**. The abundance of Actinobacteria in the colonic chyme of the *Pla2g10* KI group had an upward trend compared with the control group, whereas the abundance of Bacteroidetes was significantly lower (**Figure 5A**), and the Firmicutes/Bacteroidetes (F/B) ratio also showed an increasing trend in the *Pla2g10* KI mice (**Supplementary Figure 1B**). At the genus level, the relative abundances of *Dubosiella* (*P* = 0.020), *Enterorhabdus* (*P* = 0.030), *Lactococcus* (*P* = 0.033) and *Lactobacillus* (*P* = 0.045) were significantly elevated in the *Pla2g10* KI mice compared to control group (**Figure 5B**), while *Faecalibaculum* only showed an increasing trend (*P* = 0.066). One genus, *Allobaculum*, showed a significant decline in abundance in the gut microbiota of the *Pla2g10* KI mice compared with control mice. Differences in bacteria at other levels between the *Pla2g10* KI and control groups are shown in **Supplementary Figures 1C–E**.

**Figure 6:**
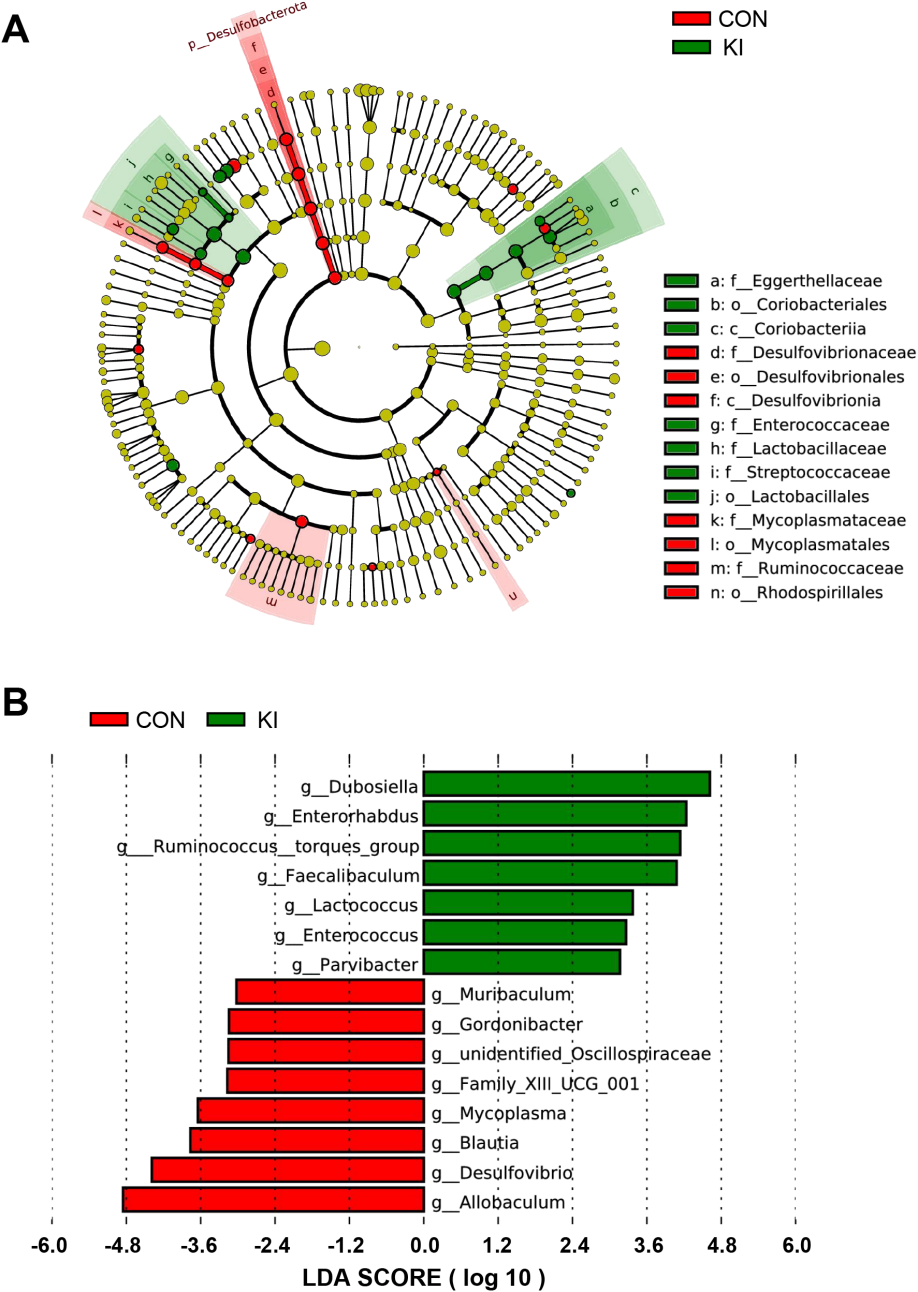
Analysis of the difference of gut microbiota between *Pla2g10* KI and control groups. **(A)** Cladogram generated by linear discriminant analysis (LDA) effect size (LEfSe) method. **(B)** LDA score reflecting the effect size of each differentially enriched taxon at genus level. CON, control group; KI, *Pla2g10* konck-in group.

To determine the effect of PLA2G10 on the gut microbial community, a linear discriminant analysis (LDA) and its effect size (LEfSe) analysis were performed to identify the biomarkers in the microbiomes of the two groups (**Figure 6A** and **Supplementary Figure 2A**). A logarithmic LDA cutoff score of 3.0 was set to identify important taxonomic differences between the two groups. We then calculated the scores that reflected the effect size of each differentially enriched taxon from these results. The LDA value histogram for the samples at the genus level (**Figure 6B**) showed that the gut microbiota of the control group contained more *Allobaculum* and *Desulfovibrio* than that of the *Pla2g10* KI group, whereas *Dubosiella and Enterorhabdus* were dominant in the *Pla2g10* KI group. Taken together, these results suggest that *Pla2g10* overexpression altered the abundances of the specific gut bacteria.

### 3.3 Impact of *Pla2g10* overexpression on microbial function

**Figure 7:**
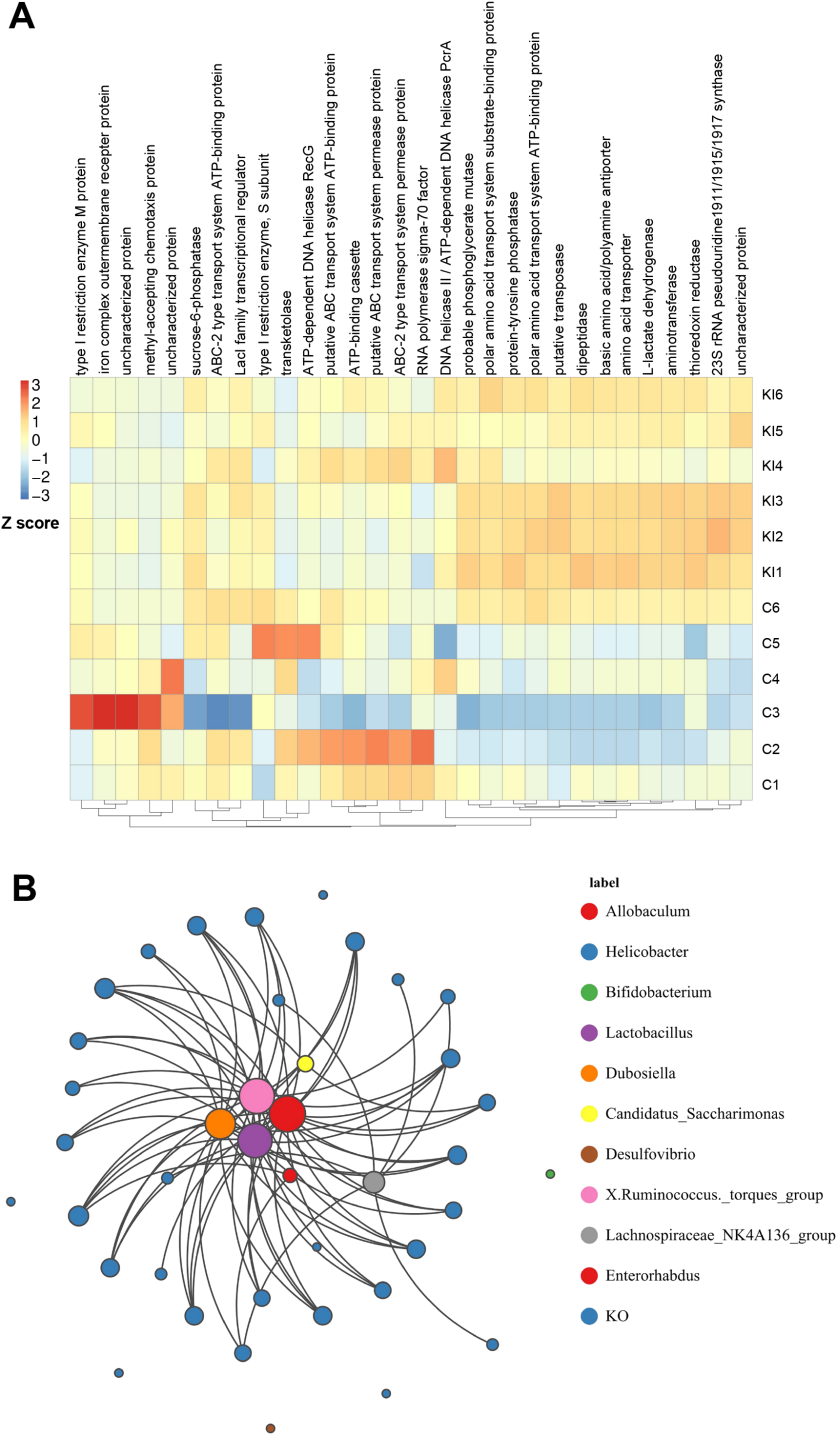
Functional prediction analysis of the gut microbiota based on the KEGG orthology database. **(A)** PICRUSt2 functional clustering heatmap of top 30 KO between two groups. **(B)** Network plot of the 30 most differentially abundant functions identified in the KEGG analysis and the 10 most abundant genera in both groups of mice. C, control group; KI, *Pla2g10* konck-in group.

To further investigate the changes in the metabolic functions of the gut microbiota associated with *Pla2g10* overexpression, we performed a functional prediction analysis based on the KEGG orthology (KO) database, using the PICRUSt2 software. Combination of the PICRUSt2 functional clustering heat map (**Figure 7A**) with a *t* test analysis (**Supplementary Figure 2B**) clearly showed that *Pla2g10* overexpression significantly altered various functions of the gut microbiota in mice, and most of the differentially expressed functions were more abundant in the *Pla2g10* KI mice than in the control mice, as shown in **Supplementary Table 2**. We then constructed a network plot of the 30 most differentially abundant functions identified in the KEGG analysis and the 10 most abundant genera in both groups of mice. The results revealed that the main genera whose functions were affected by *Pla2g10* overexpression in mice were *Dubosiella*, *Lactobacillus*, *X Ruminococcus_torques_group*, and *Allobaculum* (**Figure 7B**).

## 4 Discussion

In the current study, the value of body weight decreased, while value of food intake increased in the *Pla2g10* KI group compared with control group, however neither of them reached significance. It is generally believed that a high Firmicutes/Bacteroidetes (F/B) ratio improves energy absorption efficiency, leading to obesity related functional disorders (Murphy et al., 2010). The current study showed that the F/B ratio in the *Pla2g10* KI mice showed an increasing trend compared the control mice. Thus, the change of gut microbiota might play a role in the regulation of host nutrient utilization. However, further study will be performed to confirmed the effect of intestinal *Pla2g10* overexpression on nutrition metabolism.

Many recent studies have demonstrated that host’s genes significantly affect the diversity and functions of the gut microbiota, the abundance of specific taxa, and the genetic diversity of gut microorganisms (Lopera-Maya et al., 2022; Wang et al., 2016; Yang et al., 2022; Zhernakova et al., 2024). And in the present study, *Pla2g10* overexpression reduced the abundance of *Allobaculum*, but significantly increased the abundance of other beneficial bacteria, such as *Dubosiella*, *Enterorhabdus*, *Lactococcus* and *Lactobacillus* (**Figure 5**). A recent study showed that deficiency of *Pla2g10* decreased the abundance of several Clostridium species, and reduced levels of SCFAs, while these phenotypes could be reversed by dietary supplementation with ω-3 PUFAs (Sato et al., 2024). Interestingly, in this study, the abundance of SCFAs-producing bacteria decreased by *Pla2g10* knockout, while in our study, the abundance of SCFAs-producing bacteria like *Dubosiella* increased by *Pla2g10* knock-in. This indicated that host PLA2G10 might increase the *Dubosiella* abundance through producing ω-3 PUFAs.

The analysis of α-diversity indicated that *Pla2g10* overexpression significantly reduced the quantity and richness of the microbial community. This supports our speculation that other gut microbes compensated for the gut imbalance in the *Pla2g10-*overexpressing mice. The abundance of Actinobacteria in the gut of *Pla2g10* KI mice had an upward trend compared to the control group. Actinobacteria, like Firmicutes, are Gram-positive bacteria but have a higher GC content (Belizário and Napolitano, 2015). As shown in **Figure 5B**, the genus *Enterorhabdus*, which belongs to the phylum Actinobacteria, was significantly increased in the *Pla2g10* KI mice, which may explain the high abundance of Actinobacteria. It has been reported that *Enterorhabdus* plays a positive role in intestinal homeostasis and is negatively correlated with intestinal diseases such as ulcerative colitis and inflammatory bowel disease (IBD) (Cheng et al., 2023; Liu et al., 2022). *Enterorhabdus* is also closely involved in the regulation of glucose metabolism, as well as bile acid, fatty acid, and amino acid production by the gut microbiota in mice (Li et al., 2023; Wang et al., 2022). Therefore, PLA2G10 might also regulate host nutrient utilization through gut Enterorhabdus. However this will be further studied.

*Dubosiella* is a bacterium that produces short-chain fatty acids (SCFAs). It correlates positively with the production of butyrate and mediates the protective effect of movement on the development of nonalcoholic fatty liver disease (Wan et al., 2021; Ye et al., 2023). There has been relatively little research on *Dubosiella* before, but in recent years, a new species, *Dubosiella newyorkensis*, has been discovered, which balances the Treg/Th17 response through the production of propionic acid and L-lysine, thus exerting an immune regulatory effect on the gut (Zhang et al., 2024). It also has antioxidant and anti-aging capabilities superior to those of resveratrol, and improves the gut microbiota by increasing beneficial bacteria, such as *Lactobacillus*, *Bifidobacterium*, and *Akkermansia* (Liu T. H. et al., 2023). In our study, *Dubosiella* and *Lactobacillus* are positively correlated with the expression of various functional genes present in microbes, and intestinal *Pla2g10* overexpression increased the abundance of these two genera, indicating that the differences in functional gene expression of the gut microbiota in *Pla2g10* KI mice are mainly attributable to *Dubosiella* and *Lactobacillus*. Considering *Dubosiella*’s potential ability to regulate beneficial bacteria, we speculate that PLA2G10 regulates the abundance of microbes such as *Lactobacillus* by stimulating the proliferation of *Dubosiella*.

Similar to *Dubosiella*, *Allobaculum* can physiologically utilize carbohydrates to produce butyrate (Greetham et al., 2004). It has also been reported that *Allobaculum* constitutes the greatest proportion of bacteria in the gut microbiota, accounting for 40.42% of the total number of bacteria at the genus level (Wang et al., 2018), which is consistent with the results obtained in this study. Two contradictory conclusions have been drawn from research on *Allobaculum*. Some researchers believe that it can stimulate the proliferation of epithelial cells and improve the integrity of the intestinal barrier by producing volatile fatty acids (Fan et al., 2015), whereas another report showed that *Allobaculum* isolated from patients with IBD exacerbated colitis in mice and caused antigen-specific mucosal and systemic antibody responses (Rice et al., 2022). The role of Allobaculum the current study needs further study to be illustrated.

As mentioned earlier, there may be receptors for PLA2G10 on gut microbiota, and the PC has also been found to be exposed on the surfaces of some microorganisms, such as *Streptococcus pneumoneae* (de Faire and Frostegård, 2009), and these substances can be enzymatic targets of PLA2G10 secreted into the intestinal lumen. Consequently, we suspect that the changes in the gut microbiota caused by the overexpression of *Pla2g10* occur in multiple ways, but how PLA2G10 exerts its regulatory effect on the gut microbiota requires further research.

Although our experiments have demonstrated the effects of PLA2G10 on the composition and function of the gut microbiota, we did not determine the PUFAs levels in the intestinal lumen. So, it is uncertain whether PLA2G10 affects the composition of microbiota through the production of PUFA. Further sequencing of the gut microbiota is required after single-bacterial culture to detect the presence of PLA2G10 receptors on microorganisms.

## 5 Conclusion

In summary, overexpression of *Pla2g10* altered the composition of gut microbiota in mice. Although the α-diversity of the microbiome reduced in the *Pla2g10* knock-in mice, the abundance of beneficial microbes such as *Enterorhabdus*, *Dubosiella and Lactobacillus* increased, so our study provides a therapeutic target for the improvement of the gut microbiota in the future.

## Data Availability

The original contributions presented in the study are publicly available. This data can be found here: NCBI PRJNA1222569.
