# Supplementary material for: Intestinal overexpression of Pla2g10 alters the composition, diversity and function of gut microbiota in mice
Source: Front Cell Infect Microbiol. 2025 Mar 14;15:1535204. doi: 10.3389/fcimb.2025.1535204 (PMC11949945; doi:10.3389/fcimb.2025.1535204)
Supplement: Supplementary file 1 [file DataSheet1.zip › Supplementary figures 3.8.docx]

Supplementary Material

**Supplementary Figures**


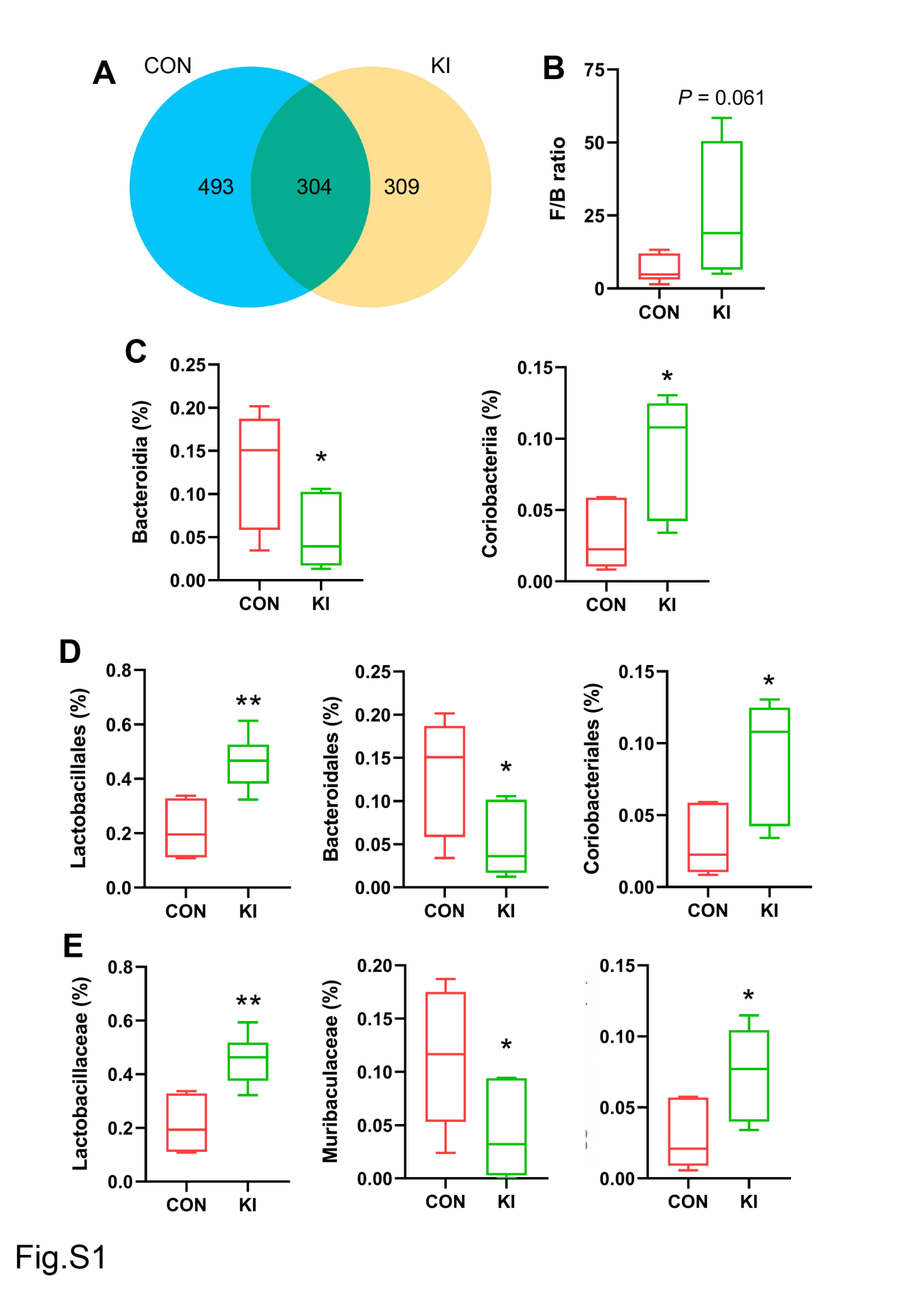


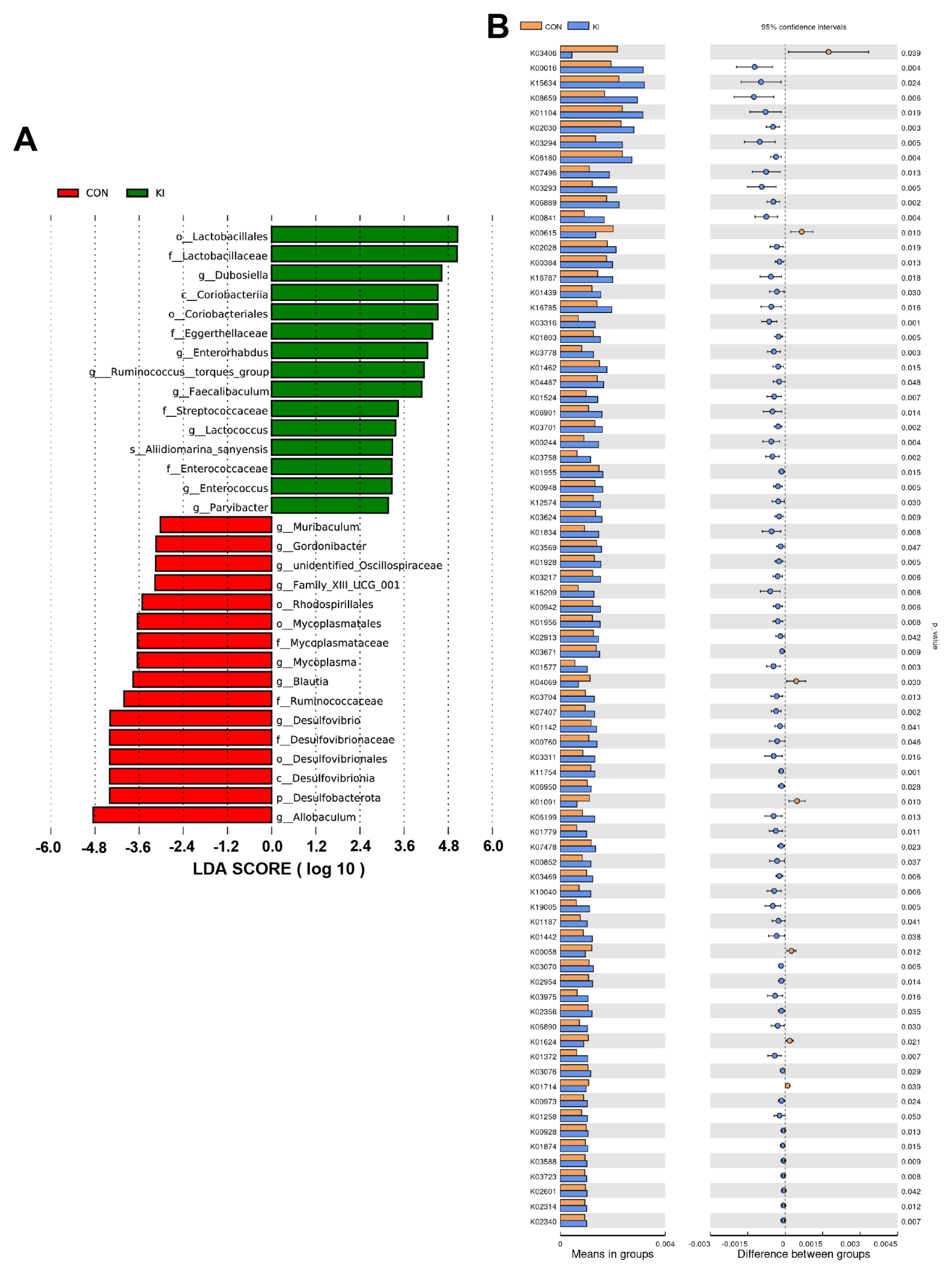


**Supplementary Figure 1.** (A) Venn diagram of OTUs between two groups. (B) The F/B ratio between the two groups. Significantly changed bacterial taxa at the class (C), order(D) and family (E).

**Supplementary Figure 2.** (A) LDA score reflecting the effect size of each differentially enriched taxon at all levels. (B) Student’s t-test was performed to analyse differential functions between the two groups. KEGG ID is a code used in the KEGG Orthology database to identify a group of genes with similar functions. An ID may represent all genes under a certain functional category, and through it, numerous genes can be classified and statistically analyzed according to their functions, revealing which functional categories are more prominent.
